# A prospective observational study to investigate the correlation analysis between neonatal hyperbilirubinemia and deafness gene: Study protocol clinical trial (SPIRIT compliant)

**DOI:** 10.1097/MD.0000000000019774

**Published:** 2020-04-24

**Authors:** Xiaohui Wu, Xingqiang Gao, Gang Li, Qiuxue Cao, Yufeng Guo, Haiyan Deng, Yun Zheng

**Affiliations:** a Department of Otolaryngology Head and Neck Surgery, Hearing Center/Hearing and Speech Science Laboratory, West China Hospital of Sichuan University; b Department of Otolaryngology-Head and Neck Surgery, Xiamen Children's Hospital; c Department of Otorhinolaryngology, Head and Neck Surgery, Minda Hospital of Hubei Minzu University, PR China.

**Keywords:** deafness, gene sequencing, neonatal hyperbilirubinemia

## Abstract

**Introduction:**

There are many studies on the relationship between serum levels of hyperbilirubinemia and hearing impairment. However, the mechanism of hyperbilirubinemia on auditory impairment is not clear.

**Methods and analysis:**

A total of 1000 children with hyperbilirubinemia who are mainly indirectly elevated bilirubin in the full-term neonatal ward of Xiamen Children's Hospital from March 2020 to September 2020 will be enrolled. Using second-generation high-throughput sequencing technology, 127 deaf-related genes were sequenced from the collected samples. At the same time, physical audiometry was performed on the selected persons and audiometry data were recorded.

**Discussion:**

In this study, we will combine gene sequencing with clinical indications of hyperbilirubinemia to find the loci suitable for high-frequency pathogenic deafness related to hyperbilirubinemia, so as to provide early guidance for deafness gene screening in children with hyperbilirubinemia.

**Trial registration:**

Chinese Clinical trial registry: ChiCTR2000030075.

## 1 Introduction

Congenital hearing impairment is the most common congenital disease, with an incidence of about 0.1% to 0.3% in healthy newborns and as high as 2% to 4% in high-risk newborns.^[1]^ The most common risk factors for neonatal hearing loss include central infection, neonatal sepsis, hyperbilirubinemia, perinatal asphyxia and hypoxic encephalopathy, preterm delivery, low birth weight, low Apgar score, prolonged mechanical ventilation, and ototoxic agents. Children with hyperbilirubinemia whose serum total bilirubin level reached the blood exchange indicator are one of the common risk factors for hearing loss in children with Neonatal intensive care unit (NICU).^[2]^ The mechanism of hyperbilirubinemia on auditory impairment is not clear. Some scholars believe that hearing loss is a prominent manifestation of bilirubin neurotoxicity.^[3]^ The brain stem auditory pathway is particularly sensitive to the toxic effect of bilirubin.^[4,5]^ High bilirubin levels can even cause permanent damage to nerve development, mainly including deafness and infant cerebral palsy.^[6,7]^

This study protocol will explore the occurrence characteristics of hearing impairment in neonatal children with hyperbilirubinemia, the relationship between serum bilirubin level, unbound bilirubin and the occurrence of hearing impairment, the mutation characteristics of deafness gene in children with hearing impairment, and the difference between deafness gene and normal population.

## 2 Methods and analysis

### 2.1 Main aims

**Figure 1:**
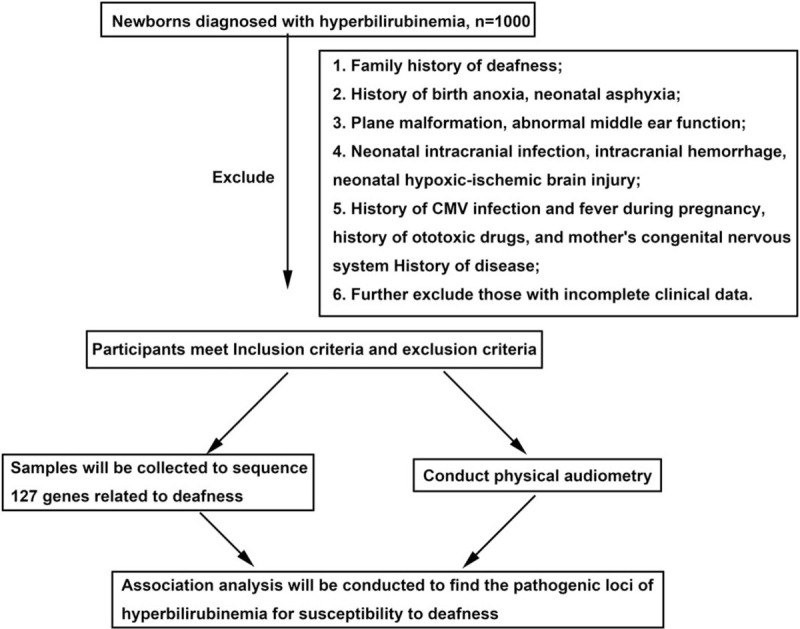
Flow chart of study protocol.

We aim to clarify the correlation analysis between neonatal hyperbilirubinemia and deafness gene. Flow chart of study protocol was shown in Figure 1.

### 2.2 Study registration

The protocol scheme matches PRISMA's reporting standards. This study protocol has been registered on Chinese Clinical trial registry (http://www.chictr.org.cn/index.aspx) with an ID of ChiCTR2000030075.

### 2.3 Participants

A total of 1000 cases of neonatal hyperbilirubinemia were diagnosed in the full-term neonatal ward of Xiamen Children's Hospital from March 2020 to September 2020.

#### 2.3.1 Inclusion criteria

The inclusion criteria are as follows:

1. Newborn ≤ 28 days, gestational age > 35 weeks, weight ≥ 1.8 kg;
2. Apgar score >7 points at 1 minute, 5 minutes, and 10 minutes after birth;
3. Neonatal who were first diagnosis as hyperbilirubinemia, not combined with elevated bilirubinemia, will receive the physical audiometry. Hearing screening was completed within 48 hours after admission: distortion product otoacoustic emission (DPOAE) and automatic auditory brainstem response (AABR) were applicable for hearing screening.

#### 2.3.2 Exclusion criteria

The exclusion criteria are as follows:

1. Family history of deafness;
2. History of birth anoxia, neonatal asphyxia;
3. Plane malformation, abnormal middle ear function;
4. Neonatal intracranial infection, intracranial hemorrhage, neonatal hypoxic-ischemic brain injury;
5. History of cytomegalovirus infection and fever during pregnancy, history of ototoxic drugs, and mother's congenital nervous system history of disease;
6. Further exclude those with incomplete clinical data.

#### 2.3.3 Diagnostic criteria for neonatal hyperbilirubinemia

According to the 2004 American academy of pediatrics guidelines for neonatal jaundice intervention, neonatal hourly bilirubinemia is defined as hyperbilirubinemia when bilirubin levels exceed the 95th percentile

### 2.4 Data collection

Neonatal patients (1000 cases) diagnosed with hyperbilirubinemia were selected, and 2 ml of blood was collected from the selected persons. Using second-generation high-throughput sequencing technology, 127 deaf-related genes were sequenced from the collected samples. At the same time, physical audiometry was performed on the selected persons and audiometry data were recorded. Genetic test data and physical audiometry data were correlated and analyzed to find the pathogenic loci of hyperbilirubinemia susceptibility to deafness. For those patients with definite symptoms of hyperbilirubinemia and deafness, and no pathogenic loci were detected in 127 genes, blood was collected from these patients, and total exon sequencing was performed to analyze about 22,000 genes and find new pathogenic loci.

### 2.5 Statistical plan

Excel was used to establish the database, and SPSS 22.0 statistical software was used for statistical analysis in this study.

1. Continuous variable data: for continuous variable data conforming to normal distribution or approximate normal distribution, mean ± standard deviation was adopted to describe the comparison of inter-group differences. When data of continuous variables do not follow normal distribution or variance is uneven, it is expressed as median (quartile spacing) M (P25, P75). Wilcoxon rank sum test in non-parametric test is performed, and multi-independent sample test is performed when there is statistical significance.
2. Categorical variables data were described as adoption rate or composition ratio of. Chi-square test was used for comparison of differences between groups, and *P* < .05 was considered statistically significant.
3. Logistic regression analysis: physical audiometry results were taken as the dependent variable, and indicators with statistical significance of univariate analysis results were taken as independent variables for binary logistic regression analysis. *P* < .05 was considered as a statistically significant difference.
4. ROC curve analysis.

### 2.6 Ethics and dissemination

This study has been approved by the ethics committee of Xiamen children's hospital. All participants’ immediate family members will sign the informed consent after being informed about the goals and methods of the study. The present study will be conducted in accordance with the tenets of the 1975 Declaration of Helsinki, as revised in 2000. The result of the study will be disseminated by publication as journal articles.

## 3 Discussion

Neonatal hyperbilirubinemia is one of the common diseases in neonatal period, especially in the first week after birth, accounting for about 50% to 75%.^[8]^ Most children have no long-term adverse effects, but hyperbilirubinemia is one of the most important risk factors for hearing loss.^[9]^ To identify and intervene the hearing loss caused by neonatal hyperbilirubinemia timely, effectively, safely, and economically in the early stage, and to reduce unnecessary treatment and waste of medical resources are the efforts of the medical community at home and abroad for many years.

Central infection, neonatal sepsis, hyperbilirubinemia, perinatal asphyxia and hypoxic-ischemic encephalopathy, preterm delivery, low birth weight, and other risk factors for neonatal hearing loss have a much higher incidence of hearing loss than common children.^[10–12]^ What are the mechanisms by which these risk factors contribute to hearing loss? Could it be that something is causing a mutation in the deafness gene that causes deafness? There are few related researches at home and abroad, which is the problem that this study aims to solve. This study protocol will explore the occurrence characteristics of hearing impairment in neonatal children with hyperbilirubinemia, the relationship between serum bilirubin level, unbound bilirubin and the occurrence of hearing impairment, the mutation characteristics of deafness gene in children with hearing impairment, and the difference between deafness gene and normal population.

## Author contributions

Xiaohui Wu conceived the idea for this study; Qiuxue Cao, Yufeng Guo and Haiyan Deng provided statistical plan; Gang Li and Yun Zheng drafted the protocol. Xingqiang Gao reviewed the protocol and provided critical feedback. All authors approved the article in its final form.
